# A Strain-Specific Inhibitor of Receptor-Bound HIV-1 Targets a Pocket near the Fusion Peptide

**DOI:** 10.1016/j.celrep.2020.108428

**Published:** 2020-11-24

**Authors:** Gabriel Ozorowski, Jonathan L. Torres, Diogo Santos-Martins, Stefano Forli, Andrew B. Ward

**Affiliations:** 1 Department of Integrative Structural and Computational Biology, The Scripps Research Institute, La Jolla, CA 92037, USA; 2 Center for HIV/AIDS Vaccine Immunology and Immunogen Discovery, International AIDS Vaccine Initiative Neutralizing Antibody Center, and Collaboration for AIDS Vaccine Discovery, The Scripps Research Institute, La Jolla, CA 92037, USA

**Keywords:** HIV-1, envelope glycoprotein, viral fusion, fusion inhibitors, cryo-EM, virtual screening, docking, small molecules, structure-based drug discovery

## Abstract

Disruption of viral fusion represents a viable, albeit under-explored, target for HIV therapeutics. Here, while studying the receptor-bound envelope glycoprotein conformation by cryoelectron microscopy (cryo-EM), we identify a pocket near the base of the trimer containing a bound detergent molecule and perform *in silico* drug screening by using a library of drug-like and commercially available molecules. After down-selection, we solve cryo-EM structures that validate the binding of two small molecule hits in very similar manners to the predicted binding poses, including interactions with aromatic residues within the fusion peptide. One of the molecules demonstrates low micromolar inhibition of the autologous virus by using a very rare phenylalanine in the fusion peptide and stabilizing the surrounding region. This work demonstrates that small molecules can target the fusion process, providing an additional target for anti-HIV therapeutics, and highlights the need to explore how fusion peptide sequence variations affect receptor-mediated conformational states across diverse HIV strains.

## Highlights

• An exogenous detergent molecule reveals a pocket in receptor-bound HIV-1 Env
• Virtual screening predicts drug-like molecules that bind the same pocket
• Cryo-EM reveals the binding of two small molecules near the fusion peptide
• One molecule inhibits the autologous HIV virus

## Graphical Abstract

**Figure:**
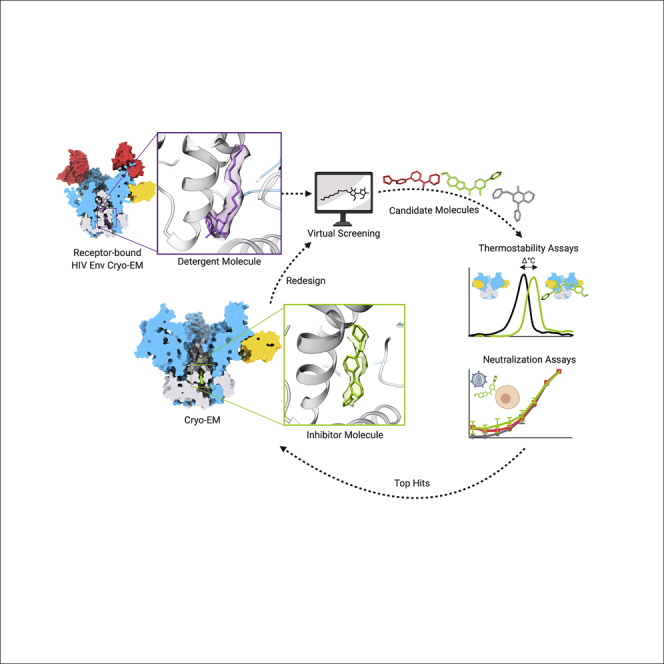

## Teaser

A limited number of HIV therapeutics target the viral Env glycoprotein. Ozorowski et al. show that a potentially druggable pocket exists in the receptor-bound form of Env and demonstrate inhibition by a small molecule, possibly due to modulation of the fusion peptide.

## Introduction

Despite advances in the characterization of HIV and treatment of infected individuals, both a functional cure and prophylactic vaccine are lacking (Andrabi et al., 2018; Davenport et al., 2019). This situation almost ensures that the global number of HIV-infected individuals will continue to rise, even under the most aggressive efforts from the medical community that have partially succeeded in slowing down the annual rate of infection (https://www.who.int/hiv/data/en/). Viremia in HIV-positive individuals can be well controlled using antiretroviral therapy (ART), which provides a relatively high quality of life by halting the progression to AIDS (Danforth et al., 2017). Furthermore, proper ART decreases HIV transmission and will continue to have a major role in fighting the HIV pandemic. Current ART methods use small molecule drugs; however, recently, a new class of potential HIV therapeutics, broadly neutralizing antibodies, has also been shown to suppress viremia in infected individuals (Barin and Braibant, 2019). The elicitation of such antibodies is the ultimate goal of HIV vaccine efforts, and the utility of recombinantly expressed versions of these antibodies for prophylaxis and ART continues to be heavily investigated. Approved ART drugs target either HIV-specific enzymes (reverse transcriptase, protease, and integrase), HIV fusion, or HIV receptors/co-receptors (CD4 and CCR5) (https://hivinfo.nih.gov/understanding-hiv/fact-sheets/fda-approved-hiv-medicines). Of the currently over three dozen Food and Drug Administration (FDA)-approved HIV medicines, only one, enfuvirtide, is a fusion inhibitor. Because viral fusion to the host cell is a necessary and conserved first step of HIV infection, the discovery of new inhibitors may lead to better ARTs that are less prone to drug resistance.

HIV fusion is facilitated by the viral envelope glycoprotein (Env), a trimer of non-covalently linked heterodimers (gp120 and gp41) (Harrison, 2015; Lee et al., 2016). Binding of the receptor CD4 to gp120 triggers a series of conformational changes, including opening of the trimer, exposure of the co-receptor binding sites, and rearrangements of the gp41 helices (Blumenthal et al., 2012; Harrison, 2015; Ozorowski et al., 2017). The N-terminal region of gp41 forms the fusion peptide (FP), which becomes sequestered during the initial steps of receptor binding by moving toward the trimer interior (Ozorowski et al., 2017). After receptor and co-receptor binding, the trimer is thought to undergo even more major conformational changes, such as gp120 shedding and the formation of the 6-helix bundle, eventually leading to the insertion of the FP into the host membrane and fusion with the viral membrane (Harrison, 2015). The FDA-approved fusion inhibitor enfuvirtide is a peptide drug mimetic that resembles a portion of the HR2 helix of gp41 and is thought to disrupt one of the penultimate gp41 changes prior to membrane fusion (Lalezari et al., 2003). Other reported small molecules that target HIV fusion and have demonstrated inhibitory activity bind instead to the closed, pre-receptor engagement state of the Env trimer, such as candidate drug molecules that are based on the Bristol Myers Squibb inhibitor BMS-626529 (Nowicka-Sans et al., 2012). In recent years, these molecules have generated excitement, with many published structures and safety and efficacy reports, as well as ongoing phase III clinical trials (Lai et al., 2019; Nowicka-Sans et al., 2012). These molecules work by binding the pre-fusion, receptor-free states of Env and halt conformational changes associated with receptor binding (Pancera et al., 2017).

Advances in cryoelectron microscopy (cryo-EM), a plethora of anti-HIV antibodies, and synthetic ligands now provide tools for structural elucidation of various Env conformations. These transient states represent new targets for small molecule inhibitors, similar to what has been done for G protein-coupled receptors (GPCRs) (Hua et al., 2017; Zheng et al., 2016). Here, we identified a pocket in gp41 of our previous cryo-EM reconstruction of an early pre-fusion intermediate Env (CD4 bound and co-receptor mimic antibody bound) (Ozorowski et al., 2017) that is proximal to the FP and contains a bound detergent molecule used during cryo-EM grid preparation. Guided by this reference molecule and its interactions with residues lining the pocket, we performed *in silico* drug screening by using a library of drug-like and commercially available small molecules. Through a combination of biophysical methods, including cryo-EM, we confirmed that two of the molecules specifically bound the pocket, in very similar manners to their predicted binding poses. One molecule in particular inhibited viral entry at low micromolar levels.

## Results

### A Potentially Druggable Pocket Forms near the FP after Receptor Binding

We previously reported cryo-EM maps of SOSIP (an engineered ectodomain of HIV-1 Env) in complex with b12 or CD4/17b that demonstrated a distinct and stable conformation of the FP and FP proximal region (FPPR) upon receptor- or antibody-induced trimer opening (Ozorowski et al., 2017). In the original (C3-symmetric) CD4-bound structure, we omitted the first three residues of the FP (A512-G514) from the atomic model due to local disorder, resulting in unassigned density within the vicinity of FP/FFPR. As an attempt to better resolve this region, the data were reprocessed using newer software (Relion 3.0, Zivanov et al., 2018; and CryoSPARC version 2, Punjani et al., 2017), including template-based particle picking to extract more particles that may have been missed by the previous difference of Gaussians approach (Voss et al., 2009). Reprocessing resulted in well-resolved C3-symmetric and asymmetric reconstructions that each exceeded the Fourier shell correlation (FSC) resolution estimate of the original map (C1: 3.6 Å, C3: 3.3 Å; EMD-8713 C3 map: 3.7 Å) and allowed for better interpretation of the FP/FPPR region (Table 1; Figures S1A–S1D). We attribute most of the improvement to an increase in the number of particles in the final reconstruction (nearly 4× that of the originally published map), which was streamlined by the template-based particle picker of CryoSPARC version 2 (Punjani et al., 2017).

**Table 1:** Cryo-EM Data Collection and Modeling Statistics

Map	B41+17b+CD4+DDM (C1)	B41+17b+CD4+DDM (C3)	B41+17b+CD4 (LMNG)	B41+17b+CD4+GO35	B41+17b+CD4+GO52 (C1)	B41+17b+CD4+GO52 (C3)
EMDB	EMDB: EMD-20152	EMDB: EMD-20151	EMDB: EMD-20153	EMDB: EMD-20150	EMDB: EMD-22049	EMDB: EMD-22048

Data Collection						

Microscope	Thermo Fisher Titan Krios	Thermo Fisher Titan Krios	Thermo Fisher Titan Krios	Thermo Fisher Titan Krios	Thermo Fisher Talos Arctica	Thermo Fisher Talos Arctica
Voltage (kV)	300	300	300	300	200	200
Detector	Gatan K2 Summit	Gatan K2 Summit	Gatan K2 Summit	Gatan K2 Summit	Gatan K2 Summit	Gatan K2 Summit
Recording mode	Counting	Counting	Counting	Counting	Counting	Counting
Nominal magnification	22,500	22,500	29,000	29,000	36,000	36,000
Movie micrograph pixel size (Å)	1.31	1.31	1.03	1.03	1.15	1.15
Dose rate (e^−^/[(camera pixel)^∗^s])	9.95	9.95	4.36	5.07	4.26	4.26
Number of frames per movie micrograph	50	50	50	50	46	46
Frame exposure time (ms)	200	200	250	250	250	250
Movie micrograph exposure time (s)	10	10	12.5	12.5	11.5	11.5
Total dose (e^−^/Å^2^)	58	58	51	60	49	49
Defocus range (μm)	−1.0 to −2.5	−1.0 to −2.5	−0.5 to −2.5	−0.6 to −2.2	−0.5 to −2.0	−0.5 to −2.0

**EM Data Processing**						

Number of movie micrographs	1,148	1,148	5,296	1,285	2,654	2,654
Number of molecular projection images in map	183,480	183,480	100,406	30,415	228,502	228,502
Symmetry	C1	C3	C3	C3	C1	C3
Map resolution (FSC 0.143; Å)	3.7	3.4	3.8	3.5	4.0	3.6
Map sharpening B-factor (Å^2^)	−116	−137	−141	−98	−134	−118

**Number of Atoms in Deposited Model**						

gp120	9,100	9,012	9,042	8,259	8,216	8,307
gp41	3,218	3,267	3,081	2,979	3,519	3,519
sCD4	2,325	2,325	2,325	2,280	2,304	2,304
Fab Fv	5,508	5,508	5,451	0	0	5,349
glycans	1,612	1,224	981	645	533	645
other ligands	89	105	0	78	75	75
MolProbity score	0.89	0.77	0.95	1.00	0.80	0.76
Clashscore	1.51	0.88	1.5	2.20	0.72	0.48
Map correlation coefficient	0.83	0.81	0.76	0.73	0.71	0.74
EMRinger score	2.04	2.85	2.04	2.02	1.50	2.36

**RMSD from Ideal**						

Bond length (Å)	0.02	0.02	0.02	0.02	0.02	0.02
Bond angles (°)	1.80	1.78	1.77	1.77	1.68	1.69

**Ramachandran Plot**						

Favored (%)	98.09	97.97	97.71	98.00	97.69	97.53
Allowed (%)	1.87	1.91	2.17	2.00	2.31	2.47
Outliers (%)	0.04	0.12	0.12	0.00	0.00	0.00
Side chain rotamer outliers (%)	0.58	0.41	0.14	0.00	0.00	0.00
PDB	PDB: 6opp	PDB: 6opo	PDB: 6opq	PDB: 6opn	PDB: 6x5c	PDB: 6x5b

**Figure 1:**
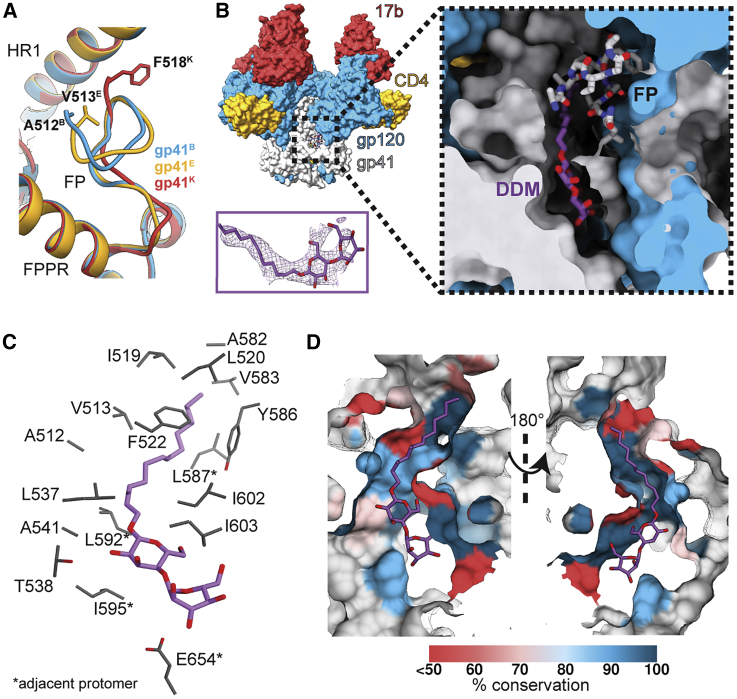
A Detergent Molecule Binds a Receptor-Induced Pocket in HIV-1 Env (A) The fusion peptide adopts different conformations in the asymmetric reconstruction of CD4- and 17b-bound B41 SOSIP. Modeled N-terminal residues of each chain are labeled. (B) Relative location of the binding pocket (left), and greater details of the locations of the DDM-containing and FP-containing pockets (right). The density for DDM in the C3-symmetry map is shown as a side panel for reference. (C and D) Contact residues (C) and percent conservation of residues (D) lining the DDM pocket. See also Figures S1 and S7.

The FP can now be fully modeled into the new C3-symmetry map and is almost completely resolved in two out of three protomers of the asymmetric map (Figure 1A). Intriguingly, both of the new maps contain additional resolved density for a long and narrow small molecule proximal to the FP in all protomers, although in the asymmetric reconstruction, this is less prominent in the protomer with a less-resolved FP (Figures 1B and S1E). Because cryo-EM freezing techniques often include sub-critical micellar concentration (CMC) amounts of detergent to increase the number and tumbling of protein particles trapped over holes in vitreous ice, we hypothesized that the unassigned density could be the DDM (n-dodecyl-β-D-maltoside) used in our experiment. In fact, two full DDM molecules and one partial DDM molecule could be built and refined into the C1 map, and C3-symmetry averaging enhances the signal for DDM in all binding sites (Figures 1B and S1E). The partial density for one of the three DDM molecules in the asymmetric reconstruction may be a result of sub-stoichiometric binding due to the low detergent concentration in the solution (approximately 2–3× molar excess of DDM to trimer), or intrinsic local asymmetry does not favor uniform binding. Recent cryo-EM reconstructions of Env SOSIP from the BG505 genotype in complex with sCD4 and a different co-receptor mimic antibody (E51) suggest asymmetry among the three protomers may be a naturally occurring feature of Env, at least in the context of receptor binding to the soluble, stabilized SOSIP construct (Yang et al., 2019). Another notable feature of the DDM-proximal residues (excluding the FP) is high conservation across HIV genotypes (Figures 1C and 1D). Hence, we further explored this pocket as a potential site for small molecule inhibitors.

### Using Cryo-EM Models for Virtual Screening

**Figure 2:**
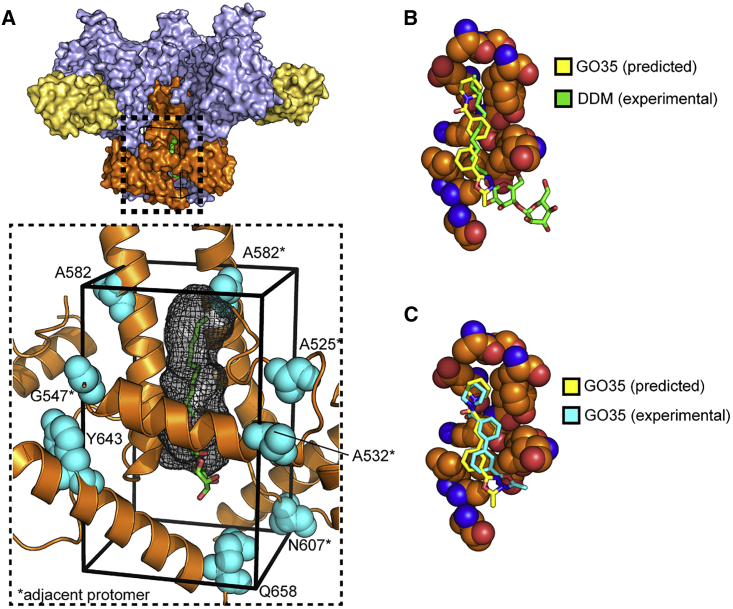
The DDM Pocket as a Template for *In Silico* Drug Screening (A) Location of the docking box with respect to the coordinates of DDM (green sticks), and the predicted AutoSite ligand binding site (black mesh); residues delimiting the box are shown as teal spheres. (B) Experimental coordinates of GO35 (yellow sticks) and DDM (green sticks) in the binding site (residues within 5 Å from any GO35 atoms as orange spheres; I519, P522, and A541 omitted for sake of clarity). (C) Experimental (yellow sticks) and docking predicted (cyan sticks) coordinates of GO35 in the binding site (residues within 5 Å from any GO35 atoms as orange spheres; I519, P522, and A541 omitted for sake of clarity). See also Figures S2 and S7 and Table S1.

The refined coordinates of DDM were used to define a ligand binding pocket to conduct *in silico* virtual screening (VS) and identify other molecules that could potentially bind. To facilitate the process, we started with the higher resolution C3-symmetric model. AutoSite (Ravindranath and Sanner, 2016) software was used to analyze the protein structure and identify the location and the size of the optimal ligand volume at the DDM binding site (Figure 2A). The docking box was centered on the AutoSite volume in 1 of the pockets (at the interface between gp120 chain A and gp41 chains B and M) and then expanded to include the larger opening engaged by the maltose moieties (with orthogonal corners roughly located between A582 and Q658) (Figure 2A). The resulting docking box was significantly larger than the reference ligand and the predicted optimal volume. This large box enabled exploration of extra hydrophilic interactions near the distal glucose ring, as well as to accommodate potential uncertainties associated with the coordinates in the cryo-EM model (e.g., decarboxylation of acidic side chains due to radiation damage; Hattne et al., 2018).

We virtually docked a library of ∼300k compounds in the pocket and applied several filters to prioritize 500 results for visual inspection. We tested the robustness of the docking protocol by repeating the VS on the three pockets in the asymmetric reconstruction, and the results highlight two major aspects: on one hand, despite structural variations resulting from the asymmetric conformational changes, the conserved topology of the pocket is sufficient to reproduce the overall ranking of the hits selected for testing using the initial C3-symmetric structure; on the other hand, the loss of interactions with FP in the more disordered conformation reduces the magnitude and range of the docking scores (Figures S2A–S2C), possibly affecting the discriminatory power of the structure in separating binders from non-binders. Therefore, we focused on compounds displaying significant overlap with DDM density from the docking results into the C3-symmetric structure and purchased 59 for further investigation.

### A Candidate Molecule Binds Near the FP and Interacts with Conserved F522

**Figure 3:**
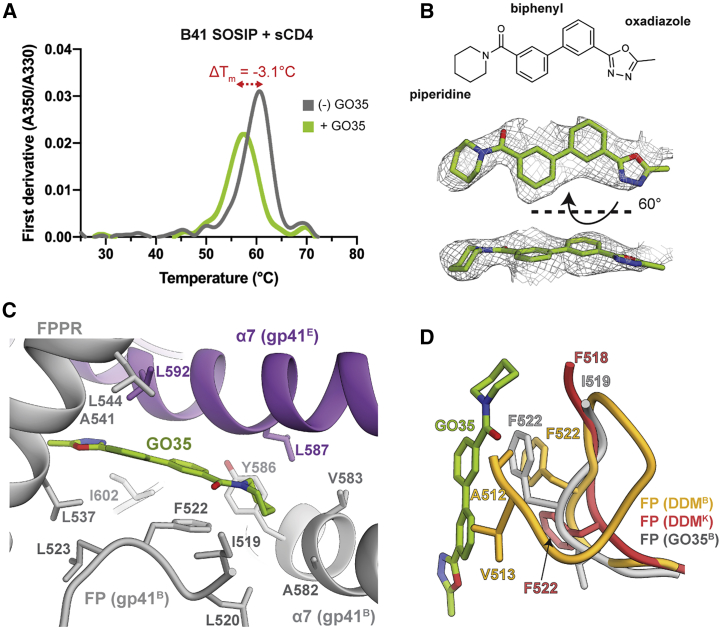
GO35 Affects the Thermostability of the Receptor-Bound Env Complex and Binds Near the Fusion Peptide (A) Differential scanning fluorimetry first derivate curves of CD4-bound B41 SOSIP in the presence or absence of GO35. (B) Chemical structure of GO35 and atomic coordinates and corresponding EM density of modeled GO35. (C) GO35 binding pocket and interaction with the fusion peptide. (D) Comparison of fusion peptides from DDM-bound and GO35-bound structures reveals that GO35 requires a different FP conformation to avoid steric clash. See also Figures S3 and S7 and Table S1.

To quickly assess the potential binding of a candidate molecule to Env SOSIP, we hypothesized that a binding event might be inferred from a change in thermostability of the protein. Previously, we showed that analogs of the known fusion inhibitor BMS-626529, which binds to gp120, significantly increased the melting temperature of SOSIP trimers (Meuser et al., 2019). We screened our compounds against six different Env SOSIP constructs (representing subtypes A, B, or C) by using differential scanning fluorimetry (DSF) by incubating a molar excess of the small molecule with a complex of SOSIP and sCD4 and by measuring the relative change in the thermal transition midpoint temperature (ΔT_m_) from a control containing the protein complex (SOSIP+sCD4) in 1% DMSO (Figure 3A; Table S1). Candidate small molecules were chosen if they met both of the following criteria: (1) a ΔT_m_ value equal or greater than ±1.0°C, and (2) reactivity against at least two different Env genotypes. Eight of the compounds had intrinsic fluorescence that interfered with the method, and from the remaining 51 candidates, 5 were selected by the above criteria (Figures S2D and S2E). Compared to assays in which sCD4 was excluded, one small molecule, GO35, stood out, as the change in T_m_ of three Env trimers was observed only in the presence of CD4, suggesting that the ligand is specific for the CD4-bound conformation (Figure 3B; Figure S2D; Table S1). This small molecule decreased the thermostability of the CD4-bound complex by about 3°C, which we inferred as a possible conformational change, and it was chosen as the first candidate for structural studies.

It was imperative that a different detergent was used for cryo-EM vitrification to decrease chances of cross-competition of DDM with candidate small molecules. DSF analysis showed that lauryl maltose neopentyl glycol (LMNG) did not have a major effect on protein stability and has twice the mass of DDM, making it unlikely to fit into the binding pocket (Figures S3A and S3B; Table S1). As a control, we solved a ∼3.7-Å cryo-EM structure of CD4- and 17b-bound B41 SOSIP frozen in the presence of LMNG (Table 1; Figures S2F and S2G). We did not see any additional density in the FP pocket that could account for detergent, and the FP itself was less ordered, similar to that of the partially bound pocket in the DDM reconstruction (Figures S2H and S2I), supporting our hypothesis that the presence of ligands affects the local stability of the FP.

Using single-particle cryo-EM, we next solved a ∼3.5-Å C3-symmetric reconstruction of a complex of B41 SOSIP, sCD4, and 17b Fab that was incubated with GO35 (Table 1; Figures S3C–S3E). The N-terminal portion of the FP is disordered until residue I519 (Figure 3C). Density for the entire GO35 molecule is sandwiched between the FPPR helix and FP of the gp41^B^ and HR1 helix of gp41^E^ (Figures 3A and 3C). One of the biphenyl rings stacks against the side chain of conserved F522 (>98% of all HIV sequences) of the FP (Figure 3C) and comprises the most extensive interaction with Env. The second ring of biphenyl is stabilized by a hydrophobic local environment consisting of L537^B^, A541^B^, L544^B^, L592^B,E^, and I602^B^. The piperidine ring of GO35 is located deepest in the pocket, although the density supports only weak hydrophobic interactions with the environment of I519^B^, L520^B^, Y586^B^, A582^B^, V583^B^, and L587^E^ (Figure 3C). On the other side of the central biphenyl is the oxadiazole ring near the entrance to the pocket, and it may form hydrogen bonds with the peptide backbone of the FPPR (Figure 3C).

Remarkably, the experimental binding mode of GO35 overlaps substantially with the position of DDM in the model used as a reference (Figure 2B) and with minimal deviation from the predicted binding mode (root-mean-square deviation [RMSD], 1.7 Å) (Figure 2C). Compared to the asymmetric DDM-containing model, the FP of the GO35-bound model is resolved only from I519, similar to the more disordered conformation seen in DDM-bound gp41 chain K, which is adjacent to a partially occupied pocket (Figure 3D). However, residues 519–525 align best with the equivalent region of DDM-bound gp41 chain B (adjacent to a fully resolved DDM molecule), particularly the side chains of F522. The full FP from the DDM-bound conformation would potentially clash with GO35 as the FP folds back on itself, bringing A512 and V513 very close to the biphenyl core of the small molecule (Figure 3D). This finding suggests that the binding of GO35 biases the FP toward the more disordered state and may force the trimer into a less stable conformation, as trimer dissociation was apparent in the cryo-EM 2D class averages, with an estimated ∼60% of the selected particles classified as individual protomers (Figure S3F).

### A Second Small Molecule Is Capable of Low Micromolar Inhibition

**Figure 4:**
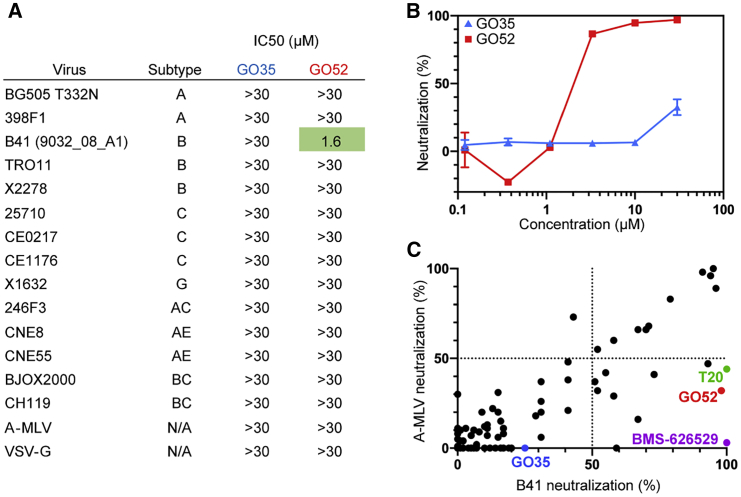
Screening for Other Hits using HIV-1 Neutralization Assays (A) Neutralization profiles of GO35 and GO52 against 14 HIV-1 and 2 control viruses. Assays were performed as duplicates (n = 2), and IC_50_s were determined by fitting an asymmetric sigmoidal five-parameter dose-response curve (R^2^ = 0.9757 for fit of GO52 against B41 virus). (B) TZM-bl neutralization curves of GO35 and GO52 against B41 pseudotyped virus. Assays were performed as duplicates (n = 2), and error bars represent standard deviation. (C) Neutralization activity of 80 small molecules against B41 HIV-1 and A-MLV viruses. All molecules were tested at a 30 μM final concentration. Assays were performed as duplicates (n = 2), and mean value is plotted. BMS-626529 and T20 (enfuvirtide) are known HIV-1 fusion inhibitors included as controls. See also Figures S4 and S7.

We next used a TZM-bl (HeLa cells engineered to express CD4, CXCR4, and CCR5) cell assay to measure whether GO35 neutralizes HIV (Figures 4A and 4B). Molecules were tested for cytotoxicity up to 60 μM, and GO35 did not have a measurable effect, so dilutions of the compound were tested in neutralization assays up to half of this value (30 μM) (Figure S4A). Despite the experimentally determined binding of GO35 to B41, neutralization was not seen against this virus nor against 13 other genetically diverse HIV-1 viruses, motivating us to further examine other small molecule hits from the virtual screen. (Figure 4A).

**Figure 5:**
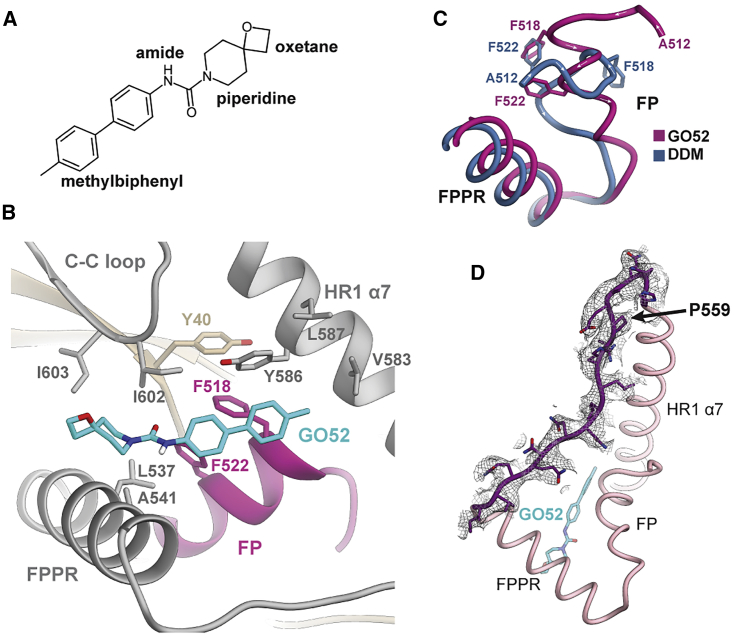
GO52 Stabilizes a New Conformation of the FP and Surrounding Regions (A) Chemical structure of GO52. (B) GO52 binding pocket and interactions with the surrounding peptide. (C) Comparison of fusion peptides from DDM-bound and GO52-bound structures in a large rearrangement in which F518 (GO52 bound) takes the place of F522 (DDM bound). (D) Model and EM map for residues 548–564 of HR1, a region that is typically disordered in published structures. See also Figures S5 and S7.

Because DSF should not be expected to pick up all binding events, nor necessarily correlate with neutralization, we screened ∼80 compounds (including analogs of GO35 and known fusion inhibitors T-20 [enfuvirtide] and BMS-626529 as positive controls) by using the TZM-bl neutralization assay against B41 and A-MLV (murine leukemia virus; negative control for HIV specificity). This screen revealed a few promising HIV specific hits, including GO52 (Figures 4C and 5A). Further assays measured an average half maximal inhibitory concentration (IC_50_) of 1.6 μM for GO52 against B41, although the small molecule was not able to neutralize other viruses in the 12-member global panel (Figures 4A and 4B). At a high concentration (30 μM), some neutralization against the negative control A-MLV was measured (Figure 4C). Interestingly, the T-20 control also showed some non-specific effects against A-MLV, but this non-specificity was not seen for BMS-626529 (Figure 4C). Due to its measured inhibition and relative specificity toward HIV-1, we next investigated GO52 further by cryo-EM.

### GO52 Binds the Trimer Base by Aromatic Interactions with gp41

During our previous cryo-EM attempts, we noticed that the complexes, particularly the trimers, had a tendency to dissociate over time in the presence of small molecules (Figure S3F). Presumably, this destabilization occurs from the presence of the solvent or even the trapping of an energetically unfavorable state of the FP. To circumvent this, we used glutaraldehyde to crosslink the B41-CD4-17b complex to stabilize it prior to small molecule addition. Indeed, fewer dissociated protomer particles were seen in the frozen samples, with an estimated 28% of particles resembling dissociated protomers compared to ∼60% in the GO35 sample (Figure S5A). Ultimately, a 3.6-Å cryo-EM reconstruction revealed that GO52 binds in the predicted pocket, and major interactions involve conserved Y586 and F522 and a rarely occurring (1.5% of sequences) F518 that is found in B41 Env (Figure 5B; Figure S5B; Table 1). The FP is reconfigured compared to the DDM-bound complex such that the side chain of F518 supplants the F522 side chain from the DDM-bound complex. F522 now becomes a secondary contact to GO52 and is trapped between F518 and L537 (of FPPR) (Figure 5C). F518 forms a cluster of five aromatic rings (F518, F522, and Y586 of gp41; Y40 of gp120; and the methylbiphenyl group of GO52) (Figure 5B).

The C3-symmetric map suggests dynamic movement of the FP centered on F522 that is not simply a difference in rotamers, so we generated an asymmetric reconstruction of the same dataset (∼4.0-Å resolution) to investigate further (Table 1; Figure S5C). In all three protomers, the phenyl group of F522 appears centered between the side chains of L537 and F518, resulting in hydrophobic and π-π stacking interactions, and the cluster of 5 aromatics is preserved (Figure S5D). The extra density near F522 in at least one protomer appears to be from hydrophobic interactions between P43 (of gp120) and the α, β, and γ carbon atoms of F522, whereas in the other two protomers, P43 interacts with either the side chains of L523 or A526 (Figure S5F). Portions of the FP in all three protomers appear to form an α-helix (Figure S5D). Symmetry expansion and focused classifications were attempted with a mask over the FP region, but we were not able to extract any additional data (including additional conformations) that was not already present in the asymmetric reconstruction.

With few exceptions, a portion of gp41 HR1 (residues 548 to 660, HXB2 numbering) is disordered in published Env structures, whether in the receptor-free or receptor-bound pre-fusion states. Sometimes the binding of a gp120-gp41 interface antibody (PGT151) confers more stability to this region or the engineering of a more stable crystal lattice (Lai et al., 2019; Lee et al., 2016). This region is also disordered in our DDM- or GO35-bound structures. Interestingly, the entire region is ordered in the GO52-bound structure and can be fully modeled (Figure 5D). Although we cannot exclude the possibility that glutaraldehyde cross-linking is responsible for this observation, the HR1 region and surrounding residues do not contain lysine residues that are most likely to be modified by glutaraldehyde (Figure S5E). Furthermore, the region is also disordered in a published BG505 Env trimer that had been crosslinked with glutaraldehyde (Schiffner et al., 2018). It is possible that increased local order is therefore a result of GO52 binding.

Although GO52 causes a global destabilization of the trimer, it does induce a relatively homogeneous and stable association between the FP, FPPR, α7 HR1 helix, and C-C loop of gp41 and C1 gp120, with possible allosteric effects on gp120 C5 (by C1 stabilization). In comparison, the C1 model of DDM-bound B41-CD4-17b exhibits greater asymmetry in this region, with F518 not playing a stabilizing role, and both F518 and F522 dramatically translocating in one of the protomers relative to the other two (Figure 3D). Furthermore, the FP is not stabilized into an α-helix in any of the protomers.

### Differences Exist between Receptor-Induced Env Rearrangements across Genotypes

**Figure 6:**
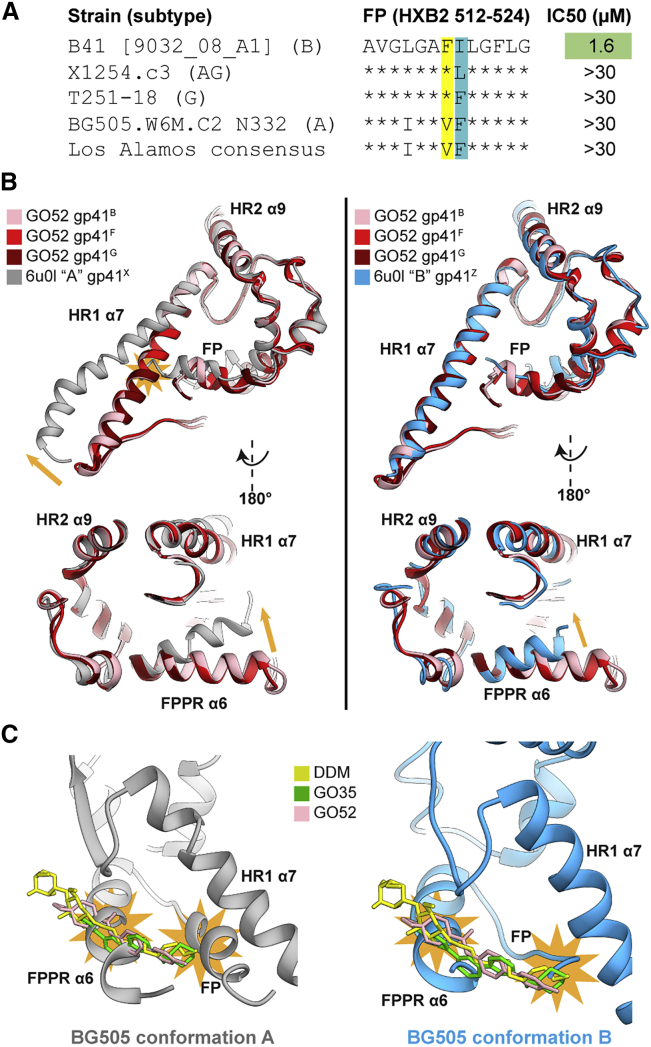
The B41 Small-Molecule Binding Pocket Is Not Amenable with Published BG505 Structures (A) Sequence alignment of the fusion peptide (HXB2, 512–524), relative to B41, of two HIV-1 sequences that also contain a phenylalanine at position 518, in comparison to BG505 and the Los Alamos consensus sequence. IC_50_s were determined as in Figure 4. (B) Overlay of CD4- and GO52-bound B41 (asymmetric) and CD4-bound BG505 conformation “A” (left) and conformation “B” (right) gp41 chains (PDB: 6u0l). Clashes denoted as orange stars; relative movements of BG505 with respect to B41 are shown as orange arrows. (C) Overlay of ligands from CD4-bound B41 with CD4-bound BG505 conformation “A” (left) and conformation “B” (right) gp41 chains. Clashes denoted as orange stars. See also Figure S6.

The specificity of GO52 against B41 prompted us to investigate whether it was due to simple amino acid variation or a larger difference of pocket accessibility across genotypes. As mentioned above, a phenylalanine residue at position 518 of the FP is rare (1.5%; 91/5,923 sequences in the Los Alamos Database). Perhaps this residue is key for neutralization, so we obtained two viruses (clade G X1254.c3 and clade AG T251-18) that naturally have F518 and only deviate from B41 by single amino acid substitutions in the FP (Figure 6A). Despite the similarity, GO52 demonstrated no neutralization activity against these pseudotyped viruses (Figure 6A).

We next hypothesized that the binding pocket may differ across genotypes and compared our CD4-bound B41 Env structures to recently published CD4-bound BG505 Env structures (that contain E51 as the co-receptor mimic antibody, instead of the 17b antibody in our structures) (Yang et al., 2019). A notable feature of the BG505 complex is that two different cryo-EM classes suggest a high degree of asymmetry between protomers. When comparing gp41 protomers from the BG505 structures to B41, we found that a shift in the relative angle of the HR1 α7-helix is apparent, in which the BG505 “conformation A protomer” helix pivots further away from the FP pocket relative to B41 (Figure 6B). The N-terminal portion of the BG505 FP clashes with the α7-helix orientation of B41. In addition, the FPPR helix (α6) is shifted more into the FP pocket in the BG505 structure and constricts the B41-derived drug pocket (Figure 6B). The repositioned FPPR and FP clash with the modeled B41 ligands (DDM, GO35, and GO52) (Figure 6C). The “conformation B protomer” has better alignment to B41, with high overlap of α7- and α9-helices, but also has the shifted FPPR helix (Figure 6B).

Our binding pocket derived from a B41 model is different than available models of BG505. The BG505 FP sequence is identical to the overall consensus sequence based on 5,923 Los Alamos HIV Database entries (Figure 6A). If the FP dictates the gp41 conformational changes, then it is possible that other genotypes have a CD4-bound structure more similar to BG505 than B41, explaining the observed specificity of GO52. In fact, when we screen our top hits derived from B41 docking against BG505 and correlate it with an A-MLV negative control, we find that none of our compounds show neutralization specificity against BG505 (Figure S4). However, even the CD4-bound BG505 data suggest conformational heterogeneity in this region (Yang et al., 2019). Although the overall maps are of high quality and resolution (3.3 and 3.5 Å overall, with no local resolutions reported that are higher than the global FSC), a comparison of the density of FPPR and surrounding regions to our DDM-bound models suggests that in BG505, this region is more flexible, as evidenced by more disorder in the maps (Figure S6).

## Discussion

The serendipitous discovery of a detergent molecule residing in a receptor-binding-induced pocket provided the framework for investigating whether it could be exploited for HIV fusion inhibitor development. Our initial hit based on binding alone, GO35, influenced the conformation of the FP by a π-π stacking interaction with a conserved phenylalanine residue but did not neutralize. GO52 showed inhibitory activity specific to the parental virus of the docking model Env B41. Comparisons to BG505 suggest that the receptor-bound conformational state varies across genotypes. It is possible that B41 naturally forms the binding pocket more often and homogenously across the three binding sites and is therefore more available for binding the small molecule. The conserved F522 residue might play a major role in regulating this state of the Env trimer. We have described before how CD4-binding site antibody b12 mimics the receptor-induced conformational changes in gp41, including those of the FP, whereas the antibody does not bind to or neutralize BG505 virus (Ozorowski et al., 2017).

We initially used a cryo-EM-derived model as a target for docking and virtual screenings, generated top candidates that were screened by using cell-based and biophysical assays, and then solved two more cryo-EM structures, each containing unambiguous evidence of small molecule binding (Figure S7). Although our efforts did not reveal molecules capable of neutralizing multiple HIV-1 strains, we did, however, succeed in finding an inhibitor that binds the specific pocket of our search model. Furthermore, the initial model has the conserved F522 in the binding pocket, whereas the rare F518 was outside of it. Thus, the resulting conformational change induced and/or trapped by GO52 came as a surprise.

Revisiting prior cryo-EM data can have broad implications. The cryo-EM field is actively evolving, and data processing techniques improve quickly. Thus, the information one can obtain from the same dataset (e.g., movie frames) has the potential to increase with innovations. Using our example, we were able to reconstruct more detailed maps simply because automated particle picking has improved since our original published structures, providing us more total particles for stringent 3D classification. Notably, advances in computation speed and available resources also have a positive influence on how many different approaches one takes in data processing.

Collectively, the results of this study not only support the viability of cryo-EM to provide data for atomistic modeling of potential drugs, which continues to be corroborated for other important drug targets (Lyu et al., 2020; Xing et al., 2020; Yin et al., 2020), but also constitute a success story of the use of molecular docking with cryo-EM structures. In fact, the approach described here has worked successfully for finding new molecules that bind to a transient target, such as an HIV pre-fusion intermediate, while capturing multiple conformational ensembles. Our results demonstrate the success of the *in silico* screens for being selective against our desired target. Future efforts will focus on elucidating the structure of this binding pocket by using another Env genotype, namely, one that has a more conserved FP sequence.

## STAR★Methods

### Key Resources Table

**Table:** 

REAGENT or RESOURCE	SOURCE	IDENTIFIER
**Antibodies**		

17b IgG fragment antigen binding	TSRI	N/A

**Bacterial and Virus Strains**		

BG505 T332N HIV-1 pseudotyped virus	TSRI	N/A
398F1 HIV-1 pseudotyped virus	TSRI	N/A
B41 (9032_08_A1) HIV-1 pseudotyped virus	TSRI	N/A
TRO11 HIV-1 pseudotyped virus	TSRI	N/A
X2278 HIV-1 pseudotyped virus	TSRI	N/A
25710 HIV-1 pseudotyped virus	TSRI	N/A
CE0217 HIV-1 pseudotyped virus	TSRI	N/A
CE1176 HIV-1 pseudotyped virus	TSRI	N/A
X1632 HIV-1 pseudotyped virus	TSRI	N/A
246F3 HIV-1 pseudotyped virus	TSRI	N/A
CNE8 HIV-1 pseudotyped virus	TSRI	N/A
CNE55 HIV-1 pseudotyped virus	TSRI	N/A
BJOX2000 HIV-1 pseudotyped virus	TSRI	N/A
CH119 HIV-1 pseudotyped virus	TSRI	N/A
A-MLV pseudotyped virus	TSRI	N/A
VSV-G pseudotyped virus	TSRI	N/A
X1254.c3 HIV-1 pseudotyped virus	TSRI	N/A
T215-18 HIV-1 pseudotyped virus	TSRI	N/A

**Chemicals, Peptides, and Recombinant Proteins**		

GO1	ChemBridge	Cat# 5104856
GO2	ChemBridge	Cat# 5175118
GO3	ChemBridge	Cat# 5253067
GO4	ChemBridge	Cat# 5256646
GO5	ChemBridge	Cat# 5280517
GO6	ChemBridge	Cat# 5318158
GO7	ChemBridge	Cat# 5325879
GO8	ChemBridge	Cat# 5528433
GO9	ChemBridge	Cat# 5568861
GO10	ChemBridge	Cat# 5961115
GO11	ChemBridge	Cat# 6017387
GO12	ChemBridge	Cat# 6633000
GO13	ChemBridge	Cat# 6729640
GO14	ChemBridge	Cat# 6772303
GO15	ChemBridge	Cat# 7294632
GO16	ChemBridge	Cat# 7361936
GO17	ChemBridge	Cat# 7371322
GO18	ChemBridge	Cat# 7560711
GO19	ChemBridge	Cat# 7779542
GO20	ChemBridge	Cat# 7780089
GO21	ChemBridge	Cat# 7782544
GO22	ChemBridge	Cat# 7791145
GO23	ChemBridge	Cat# 7987353
GO24	ChemBridge	Cat# 9013058
GO25	ChemBridge	Cat# 9039042
GO26	ChemBridge	Cat# 9113277
GO27	ChemBridge	Cat# 9211653
GO28	ChemBridge	Cat# 9262929
GO29	ChemBridge	Cat# 9333943
GO30	ChemBridge	Cat# 11331631
GO31	ChemBridge	Cat# 15275507
GO32	ChemBridge	Cat# 17562771
GO33	ChemBridge	Cat# 17695585
GO34	ChemBridge	Cat# 17932576
GO35	ChemBridge	Cat# 18983273
GO36	ChemBridge	Cat# 20845670
GO37	ChemBridge	Cat# 30439308
GO38	ChemBridge	Cat# 31886853
GO39	ChemBridge	Cat# 35911407
GO40	ChemBridge	Cat# 35921138
GO41	ChemBridge	Cat# 36550513
GO42	ChemBridge	Cat# 43044876
GO43	ChemBridge	Cat# 43130644
GO44	ChemBridge	Cat# 43390089
GO45	ChemBridge	Cat# 44220409
GO46	ChemBridge	Cat# 45532562
GO47	ChemBridge	Cat# 54662403
GO48	ChemBridge	Cat# 56393660
GO49	ChemBridge	Cat# 59973734
GO50	ChemBridge	Cat# 69927119
GO51	ChemBridge	Cat# 70997463
GO52	ChemBridge	Cat# 71013881
GO53	ChemBridge	Cat# 73880124
GO54	ChemBridge	Cat# 74873425
GO55	ChemBridge	Cat# 86846254
GO56	ChemBridge	Cat# 95796133
GO57	ChemBridge	Cat# 95872058
GO58	ChemBridge	Cat# 96194570
GO59	ChemBridge	Cat# 98689158
DEAE-Dextran	Millipore Sigma	Cat# 93556
DMEM (high glucose with L-glutamine and pyruvate)	ThermoFisher Scientific	Cat# 11995
Uranyl formate	Electron Microscopy Sciences	Cat# 22450
Dimethyl sulfoxide (DMSO)	Millipore Sigma	Cat# D8418
BMS-626529	APExBIO	Cat# A3253
T20 (enfuvirtide)	Millipore Sigma	Cat# SML0934
Dulbecco’s Phosphate buffered saline (DPBS)	ThermoFisher Scientific	Cat# 14040-182
AMC011 v4.2 SOSIP.664	TSRI	N/A
BG505 SOSIP.664	TSRI	N/A
B41 SOSIP.664	TSRI	N/A
CZA97 SOSIP.664	TSRI	N/A
DU422 SOSIP.664	TSRI	N/A
JRFL SOSIP.664	TSRI	N/A
Sodium acetate	Millipore Sigma	Cat# 241245
Tris buffered saline (TBS) 10X pH 7.4	Alfa Aesar	Cat# J60764
Soluble CD4 (Two-domain; sCD4)	TSRI	N/A
ExpiFectamine CHO Transfection Kit	ThermoFisher Scientific	Cat# A29129
Glutaraldehyde solution	MP Biomedicals	Cat# 198595
Tris Proteomics Grade	VWR Life Science	Cat# M151
Lauryl maltose-neopentyl glycol (LMNG)	Anatrace	Cat# NG310
Gentamycin	Millipore Sigma	Cat# G1272
HEPES 1M Buffer	ThermoFisher Scientific	Cat# 15630106
Fetal Bovine Serum (Heat inactivated)	ThermoFisher Scientific	Cat# 10082-147

**Critical Commercial Assays**		

CellTiter-Glo 2.0 Cell Viability Assay	Promega	Cat# G9241
Britelite Plus Reporter Gene Assay System	PerkinElmer	Cat# 6066766

**Deposited Data**		

B41 in complex with sCD4, 17b Fab and DDM (C1 symmetry)	This paper	EMDB: EMD-20152; PDB 6opp
B41 in complex with sCD4, 17b Fab and DDM (C3 symmetry)	This paper	EMDB: EMD-20151; PDB 6opo
B41 in complex with sCD4 and 17b Fab (frozen with LMNG)	This paper	EMDB: EMD-20153; PDB 6opq
B41 in complex with sCD4, 17b Fab and small molecule GO35	This paper	EMDB: EMD-20150; PDB 6opn
B41 in complex with sCD4, 17b Fab and small molecule GO52 (C1 symmetry)	This paper	EMDB: EMD-22049; PDB 6x5c
B41 in complex with sCD4, 17b Fab and small molecule GO52 (C3 symmetry)	This paper	EMDB: EMD-22048; PDB 6x5b

**Experimental Models: Cell Lines**		

FreeStyle HEK293F	ThermoFisher Scientific	Cat# R79007
ExpiCHO	ThermoFisher Scientific	Cat# A29133
TZM-bl	NIH AIDS Reagent Program	Cat# 8129
HEK293T/17	ATCC	Cat# CRL-11268

**Recombinant DNA**		

pPPI4 AMC011 v4.2 SOSIP.664	van Gils et al., 2016	N/A
pPPI4 BG505 SOSIP.664	Sanders et al., 2013	N/A
pPPI4 B41 SOSIP.664	Pugach et al., 2015	N/A
pPPI4 CZA97 SOSIP.664	Ringe et al., 2015	N/A
pPPI4 DU422 SOSIP.664	Julien et al., 2015	N/A
pPPI4 JRFL SOSIP.664	TSRI	N/A
Furin expression vector	Pugach et al., 2015	N/A
17b IgG light chain expression vector	Ozorowski et al., 2017	N/A
17b IgG heavy chain Fab expression vector	Ozorowski et al., 2017	N/A
Soluble CD4 (two-domain)	Ozorowski et al., 2017	N/A
HIV-1 NL4-3 ΔEnv luciferase reporter vector	NIH AIDS Reagent Program	Cat# 3418
pBG505 T332N HIV-1 *env*	Sanders et al., 2013	GenBank: ABA61516
p398F1 HIV-1 *env*	NIH AIDS Reagent Program	Cat# 12652
pB41 (9032_08_A1) HIV-1 *env*	Pugach et al., 2015	GenBank: EU576114
pTRO11 HIV-1 *env*	NIH AIDS Reagent Program	Cat# 11023
pX2278 HIV-1 *env*	NIH AIDS Reagent Program	Cat# 12654
p25710 HIV-1 *env*	NIH AIDS Reagent Program	Cat# 11505
pCE0217 HIV-1 *env*	NIH AIDS Reagent Program	Cat# 12660
pCE1176 HIV-1 *env*	NIH AIDS Reagent Program	Cat# 12657
pX1632 HIV-1 *env*	NIH AIDS Reagent Program	Cat# 12656
p246F3 HIV-1 *env*	NIH AIDS Reagent Program	Cat# 12658
pCNE8 HIV-1 *env*	NIH AIDS Reagent Program	Cat# 12653
pCNE55 HIV-1 *env*	NIH AIDS Reagent Program	Cat# 12661
pBJOX2000 HIV-1 *env*	NIH AIDS Reagent Program	Cat# 12655
pCH119 HIV-1 *env*	NIH AIDS Reagent Program	Cat# 12659
pSV-A-MLV *env*	NIH AIDS Reagent Program	Cat# 1065
pHEF-VSVG *env*	NIH AIDS Reagent Program	Cat# 4693
pX1254.c3 HIV-1 *env*	NIH/VRC	N/A
pT215-18 HIV-1 *env*	NIH AIDS Reagent Program	Cat# 11595

**Software and Algorithms**		

AutoSite	Ravindranath and Sanner, 2016	http://autodock.scripps.edu
ZINC	Irwin et al., 2012	https://zinc.docking.org
AutoDock Raccoon2	Forli et al., 2016	http://autodock.scripps.edu
AutoDock Vina v1.1	Trott and Olson, 2010	http://autodock.scripps.edu
Reduce	Word et al., 1999	https://www.phenix-online.org
PR.ThermControl	NanoTemper	https://nanotempertech.com/prometheus-pr-thermcontrol-software/
Gen5	BioTek	https://www.biotek.com/products/software-robotics-software/gen5-microplate-reader-and-imager-software/
GraphPad Prism version 8	GraphPad Software	https://www.graphpad.com; RRID:SCR_002798
Leginon	Suloway et al., 2005	https://sbgrid.org/software/titles/leginon
Appion	Lander et al., 2009	https://emg.nysbc.org/redmine/projects/appion
DogPicker	Voss et al., 2009	https://emg.nysbc.org/redmine/projects/appion
MSA/MRA	Ogura et al., 2003	https://emg.nysbc.org/redmine/projects/appion
MotionCorr2	Zheng et al., 2017	https://emcore.ucsf.edu/ucsf-software; RRID:SCR_016499
cryoSPARC version 2	Punjani et al., 2017	https://cryosparc.com; RRID:SCR_016501
GCTF	Zhang, 2016	https://www2.mrc-lmb.cam.ac.uk/research/locally-developed-software/zhang-software/#gctf; RRID:SCR_016500
Relion 3.0	Zivanov et al., 2018	https://www2.mrc-lmb.cam.ac.uk/relion; RRID:SCR_016274
UCSF Chimera	Pettersen et al., 2004	http://plato.cgl.ucsf.edu/chimera/; RRID:SCR_004097
UCSF ChimeraX	Goddard et al., 2018	https://www.cgl.ucsf.edu/chimerax/; RRID:SCR_015872
REFMAC5	Vagin et al., 2004	https://www.ccp4.ac.uk; RRID:SCR_014225
COOT	Emsley et al., 2010	https://www2.mrc-lmb.cam.ac.uk/personal/pemsley/coot/; RRID:SCR_014222
Phenix eLBOW	Moriarty et al., 2009	https://www.phenix-online.org/; RRID:SCR_014224
Rosetta Relax	Conway et al., 2014	https://www.rosettacommons.org; RRID:SCR_015701
MolProbity	Williams et al., 2018	https://www.phenix-online.org/; RRID:SCR_014226
EMRinger	Barad et al., 2015	https://www.phenix-online.org
Phenix software suite	Liebschner et al., 2019	https://www.phenix-online.org

**Other**		

PGT145 immuno-affinity column	Pugach et al., 2015	N/A
HiLoad 16/600 Superdex 200 prep grade column	GE Healthcare	Cat# 28989335
CaptureSelect CH1-XL column	ThermoFisher Scientific	Cat# 494346205
Amicon Centrifugal concentrator (10 kDa MWCO)	Millipore Sigma	Cat# UFC901024
Amicon Centrifugal concentrator (100 kDa MWCO)	Millipore Sigma	Cat# UFC910024
Prometheus NT.48 Standard grade capillaries (for DSF)	NanoTemper	Cat# PR-C002
Corning Black flat bottom 96 well cell culture plate with lid	Millipore Sigma	Cat# CLS3916
Electron microscopy copper mesh grids	Electron Microscopy Sciences	Cat#EMS400-Cu
Quantifoil 1.2/1.3-200 mesh holey carbon EM grids	Electron Microscopy Sciences	Cat# Q410CR1.3
C-flat 2/2-400 mesh copper EM grids	Protochips	Cat# CF-2/2-4CU-50

### Resource Availability

#### Lead Contact

Further information and requests for resources and reagents should be directed to and will be fulfilled by the Lead Contact, Andrew Ward (andrew@scripps.edu).

#### Materials availability

This study did not generate new unique reagents.

#### Data and Code Availability

Cryo-EM reconstructions and maps have been deposited to the Protein Data Bank and EM Data Bank under the accession numbers PDB: 6OPN, PDB: 6OPO, PDB: 6OPP, PDB: 6OPQ, PDB: 6X5B, PDB: 6X5C, EMDB: EMD-20150, EMDB: EMD-20151, EMDB: EMD-20152, EMDB: EMD-20153, EMDB: EMD-22048 and EMDB: EMD-22049.

### Experimental Model and Subject Details

FreeStyle 293-F (human female) and ExpiCHO cells (Chinese hamster female) were purchased from Thermo Fisher Scientific. The cells were used directly from the commercial sources following manufacturer suggestions by growing in GIBCO FreeStyle 293 Expression medium (FreeStyle 293-F cells) or GIBCO ExpiCHO Expression medium (ExpiCHO cells). Each was incubated at 37°C in the presence of 8% CO_2_ with shaking (135 rpm). Both cell lines tested negative for mycoplasma contamination. TZM-bl (human female) cells were obtained from the NIH AIDS Reagent Program (Cat# 8129) and HEK293T/17 (human female) cells were purchased from ATCC (Cat# CRL-11268). Each adherent cell line was incubated at 37°C in the presence of 5% CO2 in DMEM medium supplemented with 10% Fetal Bovine Serum (ThermoFisher Scientific), Gentamycin solution (Millipore Sigma) and HEPES (ThermoFisher Scientific). Both cell lines tested negative for mycoplasma contamination.

### Method Details

#### Binding site analysis

AutoSite (Ravindranath and Sanner, 2016) with default settings was used to analyze the initial C3 env model and identify the optimal ideal ligand volume for the DDM site. The program predicted correctly the three DDM sites (Figure 2), with optimal volumes overlapping with the hydrophobic tail of the detergent and the first glucose ring.

#### Docking

The ChemBridge ligand library (1.3M compounds [https://www.chembridge.com/] (accessed February 2019)]) was downloaded from ZINC (Irwin et al., 2012) [http://zinc.docking.org/ (accessed August 2016)], and filtered to obtain the 90% diversity set (301k ligands). Ligands were then prepared according to the standard AutoDock protocol (Forli et al., 2016). The refined model was used to extract the coordinates used for the dockings, which included two adjacent monomers of gp-41 (chain B and E), and one monomer of gp-140 (chain A). Following the standard AutoDock protocol (Forli et al., 2016), the structure was prepared by removing all non-standard amino acids, as well as glycans (no glycosylation sites were included in the docking box). Explicit hydrogens were added with Reduce (Word et al., 1999). AutoDock Vina v.1.1 (Trott and Olson, 2010) was used to perform docking calculations using a docking box centered at coordinates x = 158.694, y = 161.962, z = 133.764, and sized 20.62 × 20.62 × 33.75 Å. Results were filtered and analyzed using AutoDock Raccoon2 (Forli et al., 2016), discarding compounds with predicted score of less than −13.6, and ligand efficiency of −0.26 or worse. Through visual inspection, 59 compounds were selected and purchased. The ZINC IDs for all 59 compounds can be found in Table S1.

#### Small Molecule Stocks

Small molecules were purchased from ChemBridge (San Diego, CA). Master stocks of the small molecules (with an approximate molecular weight of ∼330 Daltons) were created by dissolving in 100% DMSO at a concentration of 20 mg/ml (∼60,000 μM). All stocks were stored at −20°C. In addition, BMS-626529 (APExBIO; dissolved to 20 mg/mL [∼42,000 μM] in 100% DMSO) and known fusion inhibitor, T20 (enfuvirtide, dissolved in PBS to 5 mg/mL [∼1,110 μM]; Sigma Aldrich) were purchased to serve as positive controls for neutralization assays.

#### Protein Expression

AMC011 v4.2 SOSIP.664, BG505 SOSIP.664, B41 SOSIP.664, CZA97 SOSIP.664, DU422 SOSIP.664, and JRFL SOSIP.664 trimers were transiently transfected with Furin in HEK293F cells (Invitrogen) and purified with in-house made PGT145 immuno-affinity columns by flowing clarified supernatant over the column, washing with a buffer composed of 20 mM Tris pH 8.0 and 500 mM NaCl, and eluting with 3 M MgCl_2_. The protein was the buffer exchanged into TBS (50 mM Tris pH 7.4, 150 mM NaCl), concentrated, and purified in TBS over size exclusion chromatography on an AKTA Pure paired with a HiLoad 16/600 Superdex 200 prep grade column (General Electric Healthcare) using methods previously described (de Taeye et al., 2015; Ringe et al., 2017). 17b Fab was expressed in ExpiCHO cells (Invitrogen), purified using a 1 mL Thermo Capture Select column (Thermo Fisher), and eluted with 0.1 M sodium acetate pH 3.5. Fractions of interest were pooled, concentrated, and buffer exchanged into TBS, with a 10 kDa concentrator (Millipore Sigma). Soluble CD4 was transiently transfected in ExpiCHO using ExpiFectamine, expressed for 14 days using the Max Titer Protocol, and purified as described previously using Ni-NTA affinity capture followed by size exclusion chromatography (Ozorowski et al., 2017). The final buffer for all protein samples was TBS.

#### Nano-Differential Scanning Fluorimetry (DSF)

Thermostability tests were performed using a nano-DSF Prometheus NT.48 instrument and standard grade capillaries (Nano-Temper Technologies). In each instance, the samples at ∼0.2 mg/ml were subjected to a temperature variance of 20°C to 95°C, using a thermal ramp of 1°C per minute. Values reported correspond to the inflection point calculated within the PR.ThermControl software (Nano-Temper Technologies).

#### Small molecule screen

A panel of 59 small molecule candidates were screened for intrinsic fluorescence. To do this, 1:100 dilutions of the small molecule master stocks were made with 1X TBS pH 7.4. The candidate molecules were resuspended in DMSO, and in all cases diluted prior to DSF experiments such that the final DMSO concentration was 1% (v/v), which was found to have a negligible impact on the melting temperature of SOSIP trimers.

#### SOSIP.664 trimers in complex with sCD4, and small molecules

10 μL of AMC011 v4.2 SOSIP.664, BG505 SOSIP.664, B41 SOSIP.664, CZA97 v3 SOSIP.664, DU422 SOSIP.664, and JRFL SOSIP.664 at 1 mg/ml were complexed with sCD4 at 1 mg/ml and each small molecule that did not show intrinsic fluorescence (GO17, 28, 30, 32, 40, 44, 47, 58 were excluded). Final concentrations of SOSIP, sCD4, and small molecules were 0.94 μM, 5 μM, and 400 μM, respectively.

#### Cytotoxicity Assays

A 3-fold dilution series of small molecules GO35 and GO52 was prepared in 100% DMSO ranging from 9,000 to 4.12 μM. 1 μL of each small molecule was added to an individual well in a black 96-well flat bottom plate, followed by 99 μL of TZM-bl cells at 0.1 million cells/ml containing 10 μg/ml DEAE-Dextran (Sigma Aldrich). Final concentrations of the small molecules were 90, 30, 10, 3.3, 1.1, 0.37, 0.12, and 0.04 μM in 1% (v/v) DMSO. Each condition was set up as duplicates. 4 reference wells containing 1% (v/v) DMSO and cells were also included, along with 4 control wells containing 1% (v/v) DMSO in DMEM media. After 2 days of incubation, 100 μL of CellTiter-Glo 2.0 Reagent (Promega) was added to each well. Plates were transferred to a Biotek Synergy H1 Microplate Reader, subjected to orbital shaking for 2 minutes followed by a 10 minute delay before luminescence was measured. Viability of the cells was calculated from values generated in the Gen5 software (Molecular Devices) by normalizing using the mean values of the DMSO and cell only wells (100% viability) and the DMSO and media only wells (0% viability). Normalization and generation of plots was performed using GraphPad Prism version 8.

#### Pseudovirus Neutralization assays

Plasmid DNA for pseudotyped viruses and TZM-bl cells were obtained from the NIH AIDS Reagent Program and as gifts from Dr. John Moore (Weill Cornell Medical College) and Mark Louder (NIH Vaccine Research Center). HEK293T/17 cells were purchased from ATCC. All pseudotyped HIV-1 Env viruses used in this study were produced by co-transfection with the NL4-3 plasmid in HEK293T/17 cells following previously described (deCamp et al., 2014) protocols and the standardized protocols of Dr. David Montefiori (https://www.hiv.lanl.gov/content/nab-reference-strains/html/home.htm). Small molecule neutralization assays were performed according to a standard TZM-bl protocol (deCamp et al., 2014) with pseudotyped HIV-1 viruses. Small molecules dissolved in DMSO were diluted 1:100 such that the final DMSO concentration was 1% (v/v), and final small molecule concentrations ranged from 0.01-30 μM. Each condition was tested in duplicate. Both virus and cell-free control plates contained 1% (v/v) DMSO. Small molecules were incubated with pseudotyped viruses for 1 hour at 37°C prior to the addition of TZM-bl cells. The calculated amount of DEAE-Dextran (Sigma Aldrich) used in the assays was 10 μg/ml. Unused wells around the perimeter of the plate were filled with DPBS to minimize evaporation of the experimental wells. After 48 hours, the Britelite Plus Reporter Gene Assay System (PerkinElmer) was added and plates were transferred to a Biotek Synergy H1 Microplate Reader and recorded using the luminescence filter and Gen5 software (Molecular Devices). Normalization was performed by calculating the mean values for reference wells containing virus, cells and 1% DMSO (0% neutralization) and the virus-free wells containing DMSO and cells (100% neutralization). Normalization, titration curves and IC_50_ calculations were performed using the GraphPad Prism software (version 8) by fitting an asymmetric sigmoidal five-parameter dose-response curve.

#### Negative stain electron microscopy

CZA97 SOSIP.664 trimers were incubated overnight at room temperature with a six-fold molar ratio of soluble CD4 and a ∼400-fold excess of small molecules GO20, 23, 34, 35, and 57. The following day, complexes were diluted to ∼0.01 mg/ml with 1X TBS pH 7.4, deposited on glow discharged copper mesh grids (Electron Microscopy Sciences), and negatively stained with 2% uranyl formate. A 120 keV FEI Tecnai Spirit with a TIETZ 4K x 4K camera was used to collect data, facilitated by the Leginon software (Suloway et al., 2005). Micrographs were stored and processed within the Appion database (Lander et al., 2009). Complexes were picked using DogPicker (Voss et al., 2009), stacked with a box size of 160 pixels, and 2D classification was performed with iterative multivariate statistical analysis/multireference alignment (MSA/MRA) (Ogura et al., 2003). Any obvious particle contaminants were removed from the classification.

#### Cryo-Electron Microscopy sample preparation

Preparation of B41+CD4+17b frozen with DDM was previously described (Ozorowski et al., 2017). For complexes of B41+CD4+17b with small molecule GO35 or GO52, ∼400 μg of B41 SOSIP.664 were incubated overnight at room temperature with sCD4 and 17b Fab, both at an approximate six-fold molar excess to SOSIP. The mixture was size-exclusion purified the following day with a HiLoad Superdex 200 pg column (GE Healthcare), and appropriate fractions were concentrated to ∼50 μL with a 100 kDa molecular weight concentrator (Amicon Ultra, Millipore). For the sample intended for incubation with GO52, an equal volume of 15 mM glutaraldehyde (Thermo Fisher Scientific) was added and incubated for 30 min, and the reaction was quenched by adding 1 M Tris (Thermo Fisher Scientific) to a final concentration of 0.1 M. Size-exclusion chromatography was performed a second time on the cross-linked sample. Final concentrations of the complexes were ∼5 mg/ml (GO35) and ∼1.2 mg/ml (GO52). Either small molecule GO35 or GO52 was diluted 1:100 into the complex (∼600 μM or 150 μM final concentrations for GO35 or GO52, respectively; ∼26x molar excess) and incubated for less than 30 minutes. To aid in particle orientation distribution, 0.5 μL of lauryl maltose-neopentyl glycol (LMNG, Anatrace) at 0.04 mM and 3.5 μL of either complex were briefly incubated prior to deposition onto Solarus plasma cleaned (Argon/Oxygen) Quantifoil 1.2/1.3-200 mesh or C-flat 2/2-400 mesh copper grids. Samples were plunge-frozen with a Thermo Fisher Vitrobot Mark IV at 10°C, 100% humidity, 10 s wait time, and a blot force of 0. The Quantifoil 1.2/1.3-200 mesh grid required an 8 s blot time, while the C-flat 2/2-400 mesh grid was blotted for 4.5 s.

#### Cryo-EM data collection and processing

Relevant map and model statistics are summarized in Table 1. All data were collected on a Titan Krios (Thermo Fisher) operating at 300 keV or Talos Arctica (Thermo Fisher) operating at 200 keV, each equipped with a Gatan K2 Summit camera. Raw micrographs were aligned and dose weighted using MotionCorr2 (Zheng et al., 2017). These micrographs were then imported into cryoSPARC version 2 (Punjani et al., 2017). CTF was estimated using GCTF (Zhang, 2016), and micrographs with a CTF fit resolution above 5 Å were discarded. Particles were picked using the template picker and were subsequently extracted with a box size of 288 pixels. Subsequent processing was continued in either cryoSPARC version 2 (Punjani et al., 2017) (DDM, LMNG, and GO35 datasets) or particles were exported to Relion 3.0 (Zivanov et al., 2018) (GO52 dataset). After numerous rounds of 2D classifications and 3D sorting, the final particles were subjected to non-uniform refinement (cryoSPARC version 2) or 3D auto-refine, CTF refinement and post processing (Relion 3.0).

#### Model Building

PDB 5VN3 (B41 SOSIP in complex with sCD4 and 17b) was used as an initial model for all datasets and fit into each respective map using UCSF Chimera (Pettersen et al., 2004). Due to masking and non-uniform refinement methods to improve resolution at the GO35-binding site, 17b Fab was excluded from the GO35- and GO52(C1)-bound models as the density for this region was of lower resolution. The coordinates for DDM were imported from the REFMAC5 (Vagin et al., 2004) dictionary in COOT (Emsley et al., 2010), while the coordinates for GO35 and GO52 were generated using Phenix eLBOW (Moriarty et al., 2009) and the ZINC (Irwin et al., 2012) SMILES string. Refinement was performed using Rosetta Relax (Conway et al., 2014) and models were validated using MolProbity (Williams et al., 2018) and EMRinger (Barad et al., 2015) included in the Phenix software suite (Liebschner et al., 2019). Figures generated using UCSF Chimera (Pettersen et al., 2004) and UCSF ChimeraX (Goddard et al., 2018).

### Quantification and Statistical Analysis

Neutralization assay curves and IC_50_ determination were using GraphPad Prism (version 8.4.2) software. All statistical measures are clearly described in the figure legends and/or in the STAR Methods.

## Supplemental Information

Document S1. Figures S1–S7 and Table S1

Document S2. Article plus Supplemental Information
