# Supplementary material for: A Strain-Specific Inhibitor of Receptor-Bound HIV-1 Targets a Pocket near the Fusion Peptide
Source: Cell Rep. 2020 Nov 24;33(8):108428. doi: 10.1016/j.celrep.2020.108428 (PMC7701285; doi:10.1016/j.celrep.2020.108428)
Supplement: Document S1. Figures S1–S7 and Table S1 [file mmc1.pdf]

Cell Reports, Volume *33*

Supplemental Information

A Strain-Specific Inhibitor

of Receptor-Bound HIV-1

Targets a Pocket near the Fusion Peptide

Gabriel Ozorowski, Jonathan L. Torres, Diogo Santos-Martins, Stefano Forli, and Andrew B. Ward

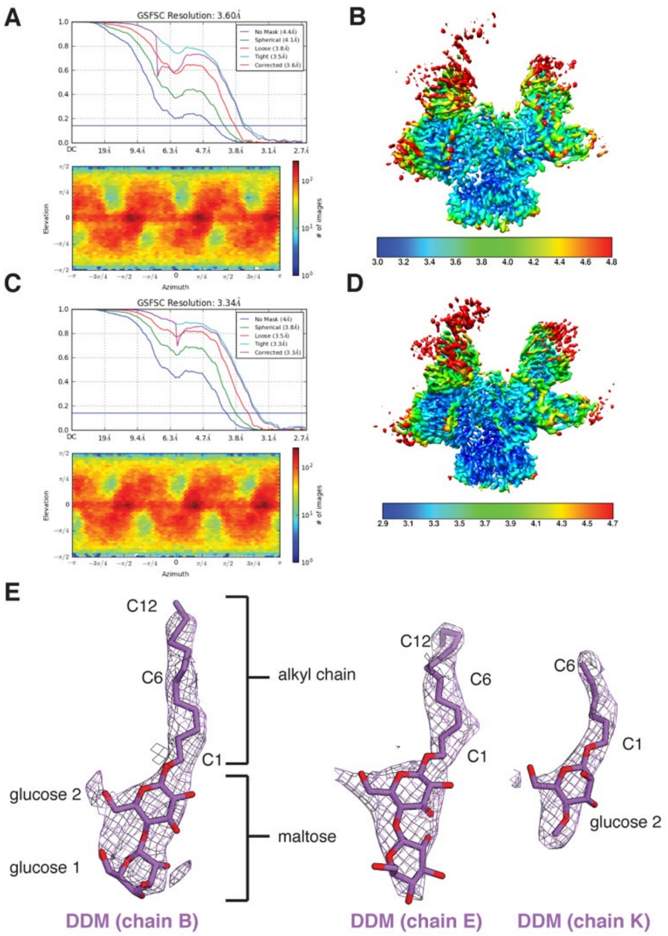

Fig. S1. Cryo-EM maps and models of detergent-bound HIV Env in complex with receptor. **Related to figure 1.** (**A**) Fourier shell correlation (*top*) and distribution plot of particle orientations (*bottom*) and (**B**) local resolution estimates (colored by Å) of C1 reconstruction of B41-CD4-17b. (**C**) Fourier shell correlation (*top*) and distribution plot of particle orientations (*bottom*) and (**D**) local resolution estimates (colored by Å) of C3 reconstruction of B41-CD4-17b. (**E**) Density and modeled atoms for DDM in three protomers of the asymmetric reconstruction of B41-CD4-17b.

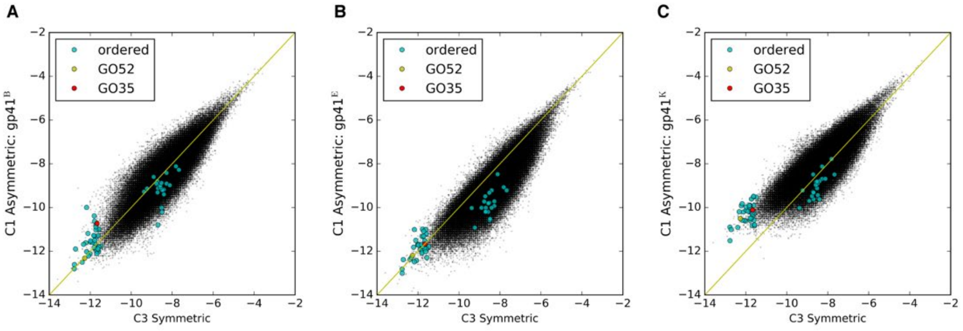

D

Small Molecule	AMC011+CD4	AMC011	BG505+CD4	BG505	B41+CD4	B41	CZA97+CD4	CZA97	DU422+CD4	DU422	JRFL+CD4	JRFL
GO20	-1.3	-1	-0.9	-0.8	-0.7	0.2	-1.2	-1.5	-1.3	-0.8	-0.8	-0.1
GO23	0.1	0.1	0.2	0.2	-0.1	0.3	-4.3	0	2.4	0.1	0	0.1
GO34	-0.5	-0.7	-0.4	-0.6	-1.1	-0.2	-1	-1.3	1.2	-0.3	-0.8	-0.4
GO35	-0.6	-1.4	-1.1	0.2	-3.1	-0.1	-1.5	-1.9	-1.1	-0.4	-1.9	0.2
GO57	-0.7	-0.5	-1	-1.1	-1	-0.2	-1.2	-1	-2	-0.8	-1.6	-1

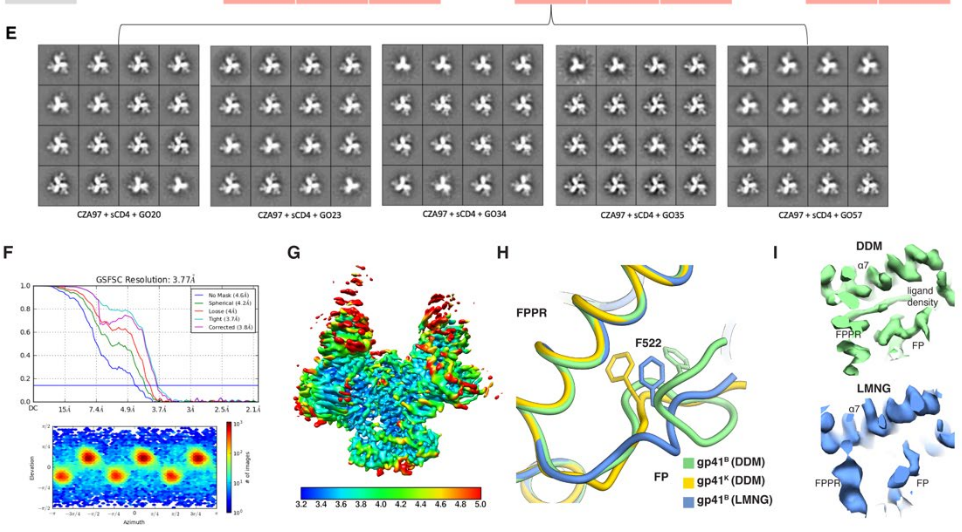

Fig. S2. Selection of small molecule candidates by *in silico* screening and differential scanning fluorimetry. **Related to figure 2.** Plot of scores obtained in the C1 asymmetric structure (y-axis) against scores obtained in the C3 symmetric structure (x-axis). Each of the three panels corresponds to a different FP conformation as represented in **Fig. 1A**: (**A**) gp41^{B}, (**B**) gp41^{E} and (**C**) gp41^{K}. (**D**) Top 5 candidates based on a selection criteria of a ΔT_m value equal or greater than ±1.0°C against at least 2 different Env genotypes, in the presence or absence of sCD4. (**E**) Negative-stain 2D class averages of CZA97 SOSIP+sCD4 complexed with the top 5 candidates in (**D**) reveals that changes in stability are not due to trimer dissociation/denaturation. (**F**) Fourier shell correlation (*top*) and distribution plot of particle orientations (*bottom*) and (**G**) local resolution estimates (colored by Å) of C3 reconstruction of B41-CD4-17b frozen with LMNG. (**H**) Comparison of fusion peptides from B41-CD4-17b frozen with either DDM (asymmetric model) or LMNG. (**I**) Comparison of EM map (contoured at 1.5σ) near the ligand binding pocket of B41-CD4-17b frozen with either DDM (green) or LMNG (blue).

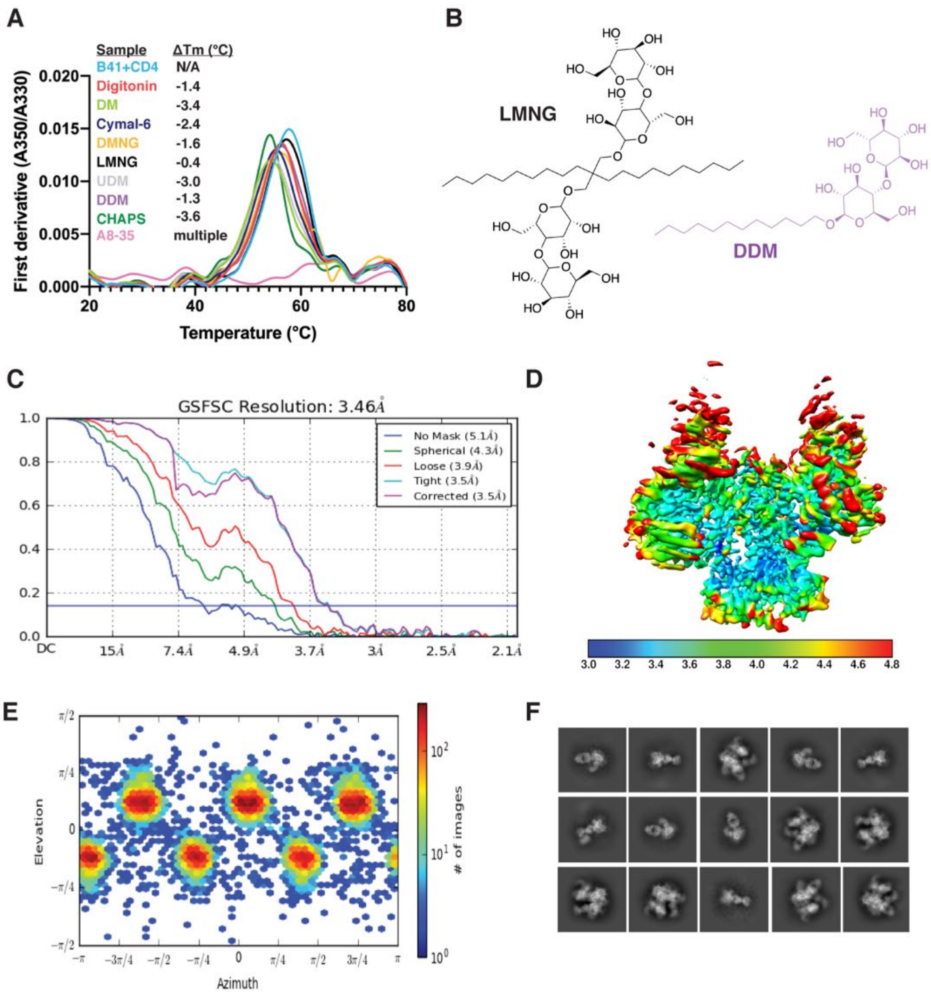

Fig. S3. Targeting the DDM-pocket of gp41 with small molecules. **Related to figure 3.** (**A**) Change in T_m of B41 SOSIP in the presence of various detergents. (**B**) Comparison of DDM and LMNG structures. (**C**) Global Fourier shell correlation (FSC), (**D**) side view of the final 3D reconstruction colored by local resolution estimates (in Å), and (**E**) distribution plot of particle orientations of B41+CD4+17b+GO35. (**F**) Select 2D class averages of B41+CD4+17b+GO35.

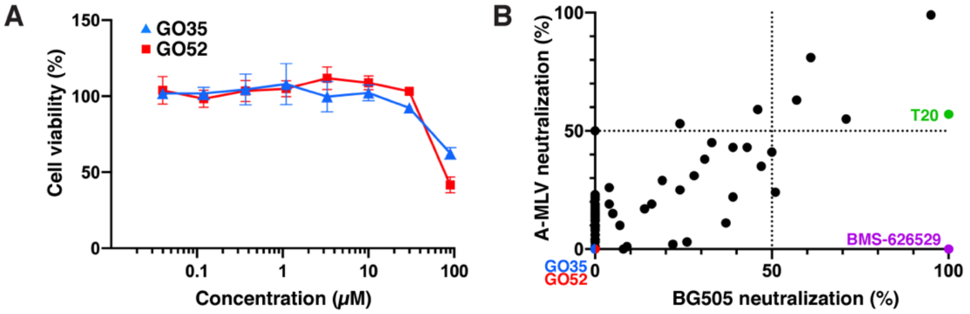

Fig. S4. **Cytotoxicity and neutralization assay profiles of small molecules. Related to figure 4.** (**A**) Cytotoxicity measurements of GO35 and GO52. Assays performed as duplicates. Mean values plotted with standard deviation represented by vertical bars. (**B**) Neutralization activity of 80 small molecules against BG505 N332 HIV-1 and A-MLV viruses. All molecules tested at 30 μM final concentration. Assay performed as duplicates (*N=2*) and mean value is plotted. BMS-626529 and T20 (enfuvirtide) are known HIV-1 fusion inhibitors included as controls.

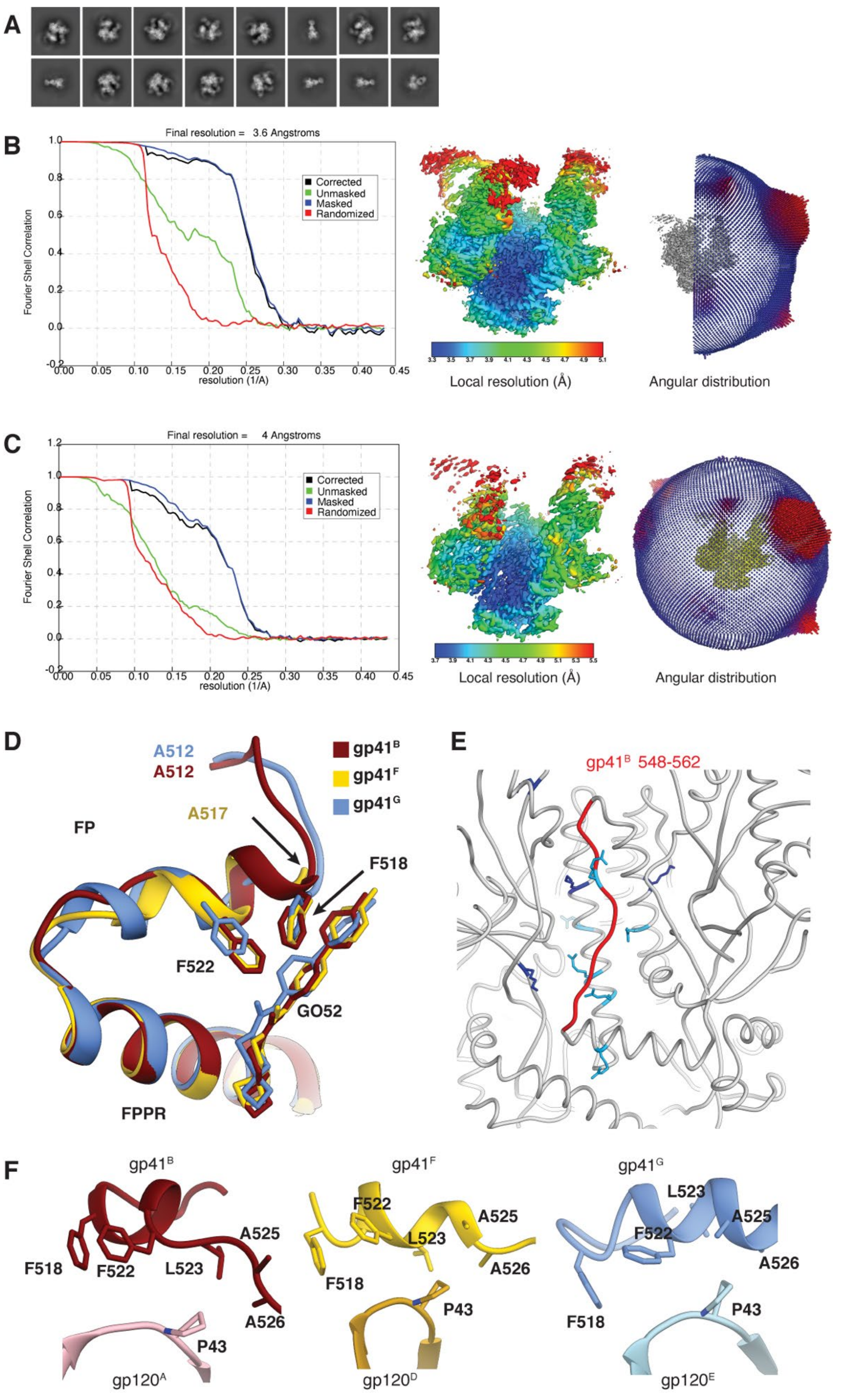
A
B
Final resolution = 3.6 Angstroms
Fourier Shell Correlation
resolution (1/A)
Corrected
Unmasked
Masked
Randomized
Local resolution (Å)
Angular distribution
C
Final resolution = 4 Angstroms
Fourier Shell Correlation
resolution (1/A)
Corrected
Unmasked
Masked
Randomized
Local resolution (Å)
Angular distribution
D
A512
A512
A517
FP
F518
F522
GO52
FPPR
gp41B
gp41F
gp41G
E
gp41B 548-562
F
gp41B
F518
F522
L523
A525
A526
P43
gp120A
gp41F
F522
A525
L523
F518
A526
P43
gp120D
gp41G
L523
A525
F522
A526
F518
P43
gp120E

Fig. S5. Cryo-EM reconstructions of B41+CD4+17b+GO52. Related to figure 5. (**A**) Select 2D class averages of B41+CD4+17b+GO52. Global Fourier shell correlations (FSC; *left*), local resolution estimates (colored by resolution in Å; *middle*) and distribution plot of particle orientations (*right*), of the (**B**) C3 symmetric and (**C**) asymmetric reconstructions of B41+CD4+17b+GO52. (**D**) Superimposition of the 3 asymmetric gp41 chains of (**C**) with a focus on the FP and FPPR regions. (**E**) Lysine (dark blue) and arginine (light blue) residues within 10 Å of gp41 HR1 region 548-562. (**F**) Comparison of P43 (gp120) interactions with the FP in the three asymmetric protomers of (**C**).

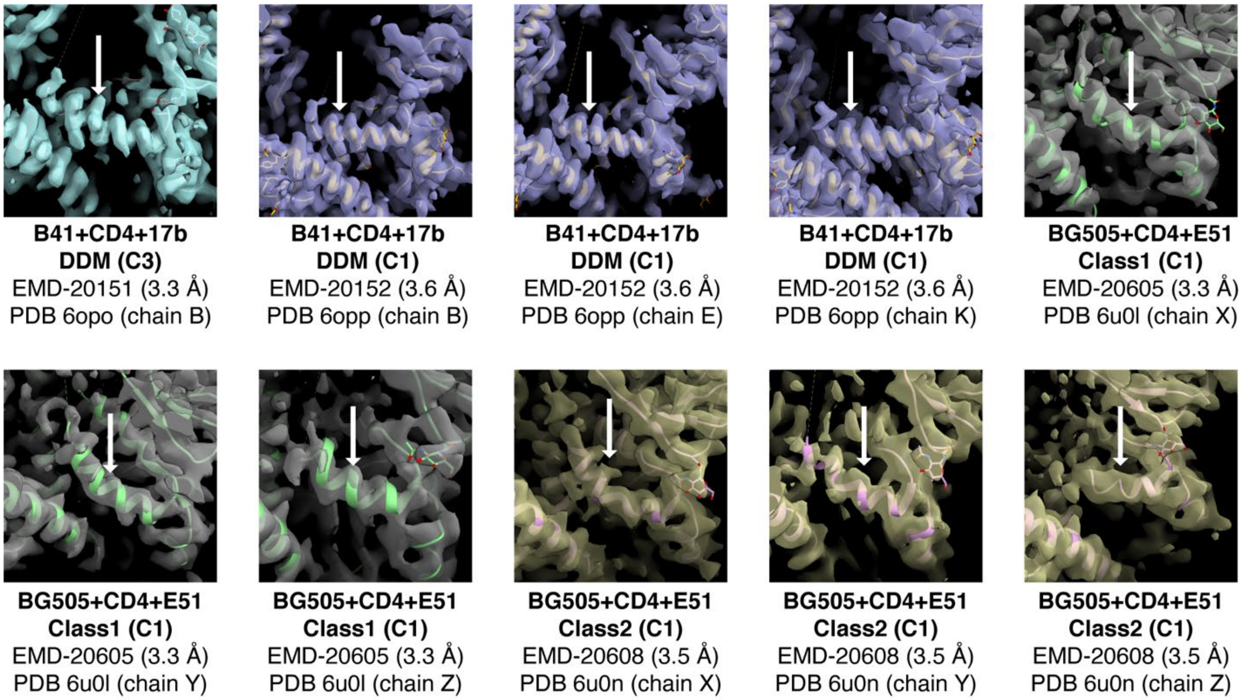

Fig. S6. The B41 binding FPPR is more ordered than in BG505. **Related to figure 6.** Comparison of CD4- and DDM-bound C3-symmetric and asymmetric B41 cryo-EM maps with asymmetric reconstructions of CD4-bound BG505, with special focus on the FPPR (denoted by a white arrow).

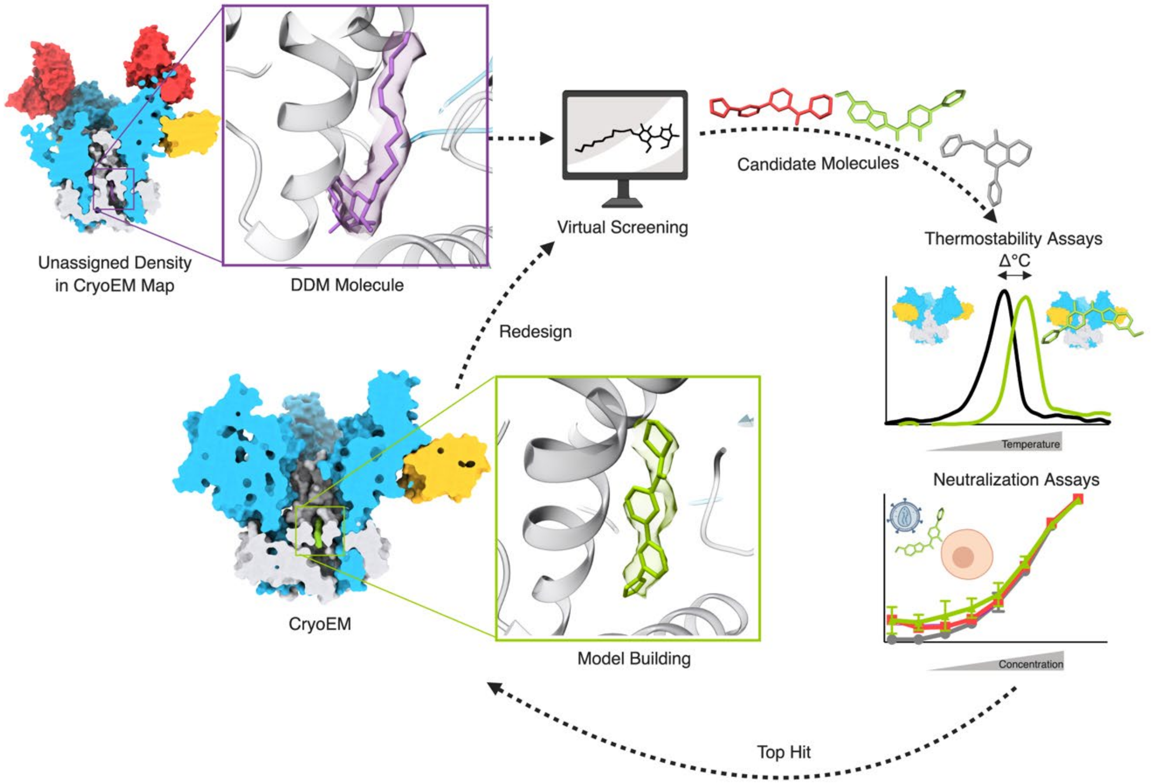

Fig. S7. **A schematic of iterative drug design using a combination of cryo-EM, virtual screening, and assays. Related to figures 1, 2, 3, 4, and 5.** Created with BioRender.com.

Table S1. Difference in thermal stability of CD4-bound SOSIP trimers in the presence of small molecules relative to no small molecule controls as measured by differential scanning fluorimetry. Related to figures 2 and 3. Absent values marked with a star denote that the small molecule had a high level of intrinsic fluorescence that interfered with the method.

Small Molecule	ZINC ID	AMC011+CD4	BG505+CD4	B41+CD4	CZA97+CD4	DU422+CD4	JRFL+CD4	Small Molecule	ZINC ID	AMC011+CD4	BG505+CD4	B41+CD4	CZA97+CD4	DU422+CD4	JRFL+CD4
**GO1**	ZINC00185349	-0.1	0.1	-0.4	-0.1	0.6	0	**GO31**	ZINC67459760	-0.4	-0.4	-0.3	-0.1	-0.3	0
**GO2**	ZINC01950659	-0.1	-0.1	-0.3	-0.5	0.6	-0.2	**GO32**	ZINC77300595	*	*	*	*	*	*
**GO3**	ZINC00235832	-0.1	0	-0.3	-0.3	0.7	-0.3	**GO33**	ZINC67491033	0.1	-0.1	-0.1	0.1	0	0
**GO4**	ZINC00291882	-0.1	0.2	-0.3	-0.3	1	-0.2	**GO34**	ZINC97244092	-0.5	-0.4	-1.1	-1	1.2	-0.8
**GO5**	ZINC00233220	-0.1	-0.1	-0.3	-0.3	1.1	-0.1	**GO35**	ZINC96132694	-0.6	-1.1	-3.1	-1.5	-1.1	-1.9
**GO6**	ZINC01907589	0.1	0.1	-0.3	0	1.1	-0.2	**GO36**	ZINC91325610	-0.4	-0.4	-0.7	-0.8	0.3	-0.8
**GO7**	ZINC03998067	0	0.1	-0.3	-0.2	0.6	-0.2	**GO37**	ZINC67675494	0	0.2	-0.4	-0.6	0.8	-0.2
**GO8**	ZINC00258817	0	0.1	-0.5	0	0.4	-0.2	**GO38**	ZINC20782685	0	-0.5	-0.7	-0.6	-0.4	-0.5
**GO9**	ZINC00035601	0.2	0	0	-0.1	0	-0.2	**GO39**	ZINC65394371	-0.2	-0.1	-0.2	0	-0.2	-0.1
**GO10**	ZINC12362428	0.1	0.1	-0.2	-0.1	0.4	-0.1	**GO40**	ZINC65394381	*	*	*	*	*	*
**GO11**	ZINC00123118	0.2	0	-0.4	-0.1	0.5	0	**GO41**	ZINC55153733	0.2	0	-0.9	-0.6	0.8	-0.4
**GO12**	ZINC18247429	0.1	0.1	-0.2	-0.2	0.5	0.2	**GO42**	ZINC71745481	-0.6	-0.7	-1.1	-0.4	-0.6	-0.7
**GO13**	ZINC00074920	0.1	-0.1	-0.2	-0.1	0.5	0.1	**GO43**	ZINC95366696	0.1	0.2	-0.4	-0.4	1.4	-0.2
**GO14**	ZINC00569594	-0.1	0.1	-0.2	-0.4	1.3	-0.2	**GO44**	ZINC77386563	*	*	*	*	*	*
**GO15**	ZINC00461274	-0.3	-0.1	-0.2	-0.1	-0.3	-0.1	**GO45**	ZINC67803542	0	0.1	-0.3	-0.4	1.1	-0.2
**GO16**	ZINC00462885	0.1	0.2	-0.2	-0.1	0.4	0	**GO46**	ZINC77389895	-0.3	-0.4	-1	-0.6	0.3	-0.7
**GO17**	ZINC00463378	*	*	*	*	*	*	**GO47**	ZINC96149848	*	*	*	*	*	*
**GO18**	ZINC00469391	-0.2	-0.1	0.1	0.1	0.2	-0.1	**GO48**	ZINC55252643	-0.2	0.1	-0.5	-0.5	0.1	-0.2
**GO19**	ZINC04263843	0.8	-0.2	-0.2	0.1	1	-0.2	**GO49**	ZINC65422611	-0.1	0	-0.1	-0.2	-0.3	-0.1
**GO20**	ZINC05059895	-1.3	-0.9	-0.7	-1.2	-1.3	-0.8	**GO50**	ZINC14974795	-0.1	-0.1	-0.7	-0.6	0.1	-0.2
**GO21**	ZINC00621192	0.2	0	-0.1	0	0.8	-0.4	**GO51**	ZINC65455118	0.1	0.4	-0.6	0	0.2	-0.1
**GO22**	ZINC05061043	0	-0.2	-0.5	-0.2	1.1	0	**GO52**	ZINC97681845	-0.3	0	-0.4	-0.7	1.3	-0.2
**GO23**	ZINC04247959	0.1	0.2	-0.1	-4.3	2.4	0	**GO53**	ZINC91819525	-0.1	-0.2	-0.8	-0.6	0.8	-0.5
**GO24**	ZINC00541458	0.2	0.2	-0.3	-0.1	1.1	0.2	**GO54**	ZINC67843155	-0.4	-0.3	-0.6	-0.2	-0.2	-0.3
**GO25**	ZINC01428810	0.2	0.2	-0.5	-0.1	0.8	0.1	**GO55**	ZINC97766602	-0.2	-0.2	-0.1	-0.5	0	-0.3
**GO26**	ZINC10461636	0.2	0.3	-0.1	-0.4	2	0.2	**GO56**	ZINC72171577	-0.4	-0.3	-0.5	-0.4	-0.2	-1.8
**GO27**	ZINC06767131	0.3	0.2	-0.2	-0.2	2.1	-0.1	**GO57**	ZINC67975161	-0.7	-1	-1	-1.2	-2	-1.6
**GO28**	ZINC36351253	*	*	*	*	*	*	**GO58**	ZINC67975538	*	*	*	*	*	*
**GO29**	ZINC36358921	0.2	0.1	-0.1	-0.1	1.9	0	**GO59**	ZINC72173974	-0.1	-0.4	-0.6	-0.7	-0.2	-0.4
**GO30**	ZINC77286437	*	*	*	*	*	*								
